# Use of intranasal esketamine in a girl with treatment-resistant depression and autism spectrum disorders: a case report

**DOI:** 10.17179/excli2022-4694

**Published:** 2022-02-28

**Authors:** Miriam Olivola, Vincenzo Arienti, Nicola Bassetti, Serena Civardi, Natascia Brondino

**Affiliations:** 1 Department of Brain and Behavioral Sciences, University of Pavia, Pavia, Italy; 2 ASST Pavia, Pavia, Italy

**Keywords:** autism spectrum disorders, depression, esketamine, treatment-resistant

## Abstract

Major depression is a common comorbidity in autism spectrum disorder (ASD), often difficult to identify and to treat. Autistic subjects are more at risk for suicidal thoughts and behaviors compared to typically developing peers. Unfortunately, ASD individuals are more frequently treatment-resistant and often show side-effects which reduce efficacy. Intranasal esketamine has been recently approved as an add-on medication for treatment-resistant depression (TRD), but it has never been used in ASD with comorbid major depression. Of note, a pilot study of intranasal ketamine has shown no effect on social withdrawal in ASD without depression. The present case report describes the first girl with ASD and comorbid TRD treated with intranasal esketamine.

## Introduction

Autism spectrum disorders (ASD) are often burdened by several other psychiatric conditions, among which major depression (MDD) is one of the most frequent (Fusar-Poli et al., 2020[4]; Steward et al., 2006[8]). It could be hard for the clinician to formulate an exact diagnosis of depression in the context of ASD, as many symptoms can overlap and, in most cases, autistic subjects may have difficulties in defining their inner states. Of note, ASD women, which are often difficult to identify due to their learning of several camouflaging techniques during the years, may require a psychiatric evaluation primarily for depression (South et al., 2020[7]). Suicide risk in autistic individuals increased in recent years: autistic women were three times more likely to die from suicide than women without ASD and young autistic subjects are twice as likely to commit suicide compared to young people without ASD (Kirby et al., 2019[6]). Pharmacological strategies for major depression are still ineffective in approximately one third of the patients and short acting treatments for suicidality are still lacking. Specifically, treatment resistant depression (TRD) is characterized by persistent depressive symptoms despite at least two trials of antidepressants at appropriate dosage and treatment duration. The lack of valid therapeutic options in the context of depression is particularly relevant in ASD, as many subjects may experience adverse side effects and low efficacy compared to typically developing depressed individuals. Recently, intranasal esketamine has been approved for treatment resistant depression after showing a rapid action on both depressive symptoms and suicidal ideation (Canuso et al., 2018[1]). Esketamine, the levo-enantiomer of Ketamine, is a non-competitive and non-selective antagonist of *N*-methyl-d-aspartate receptor (NMDAR). This unique mechanism of action can enhance glutamatergic transmission, resulting in BDNF release and increased synaptogenesis and neuroplasticity. Alterations in glutamatergic signaling are found in depression but also in ASD (Carlson, 2012[2]; Kadriu et al., 2019[5]). In this regard esketamine could be a useful tool in the treatment of both conditions (Daly et al., 2018[3]; Wink et al., 2021[9]). Intranasal ketamine (Wink et al., 2021[9]) has been already used in ASD without depression as a potential treatment for social withdrawal, without significant effect. However, only mild side-effects (fatigue, headache, nausea, transient tachycardia) were observed.

## Case Presentation

G.B. is a 24-year-old Caucasian autistic girl without intellectual disability (WAIS yielded an IQ score of 107). Her family history of psychiatric disorders was positive, as her maternal grandmother suffered from MDD and her paternal grandfather experienced panic attacks. From early childhood G. showed difficulties in her social skills and peer relationships and was therefore referred to a child psychiatrist; however, no psychiatric diagnosis was formulated during childhood. During high school she started psychotherapy, continuing nowadays. After graduating from high school, she enrolled at the university, and she is currently attending a Natural Sciences degree program. In 2019 G. was hospitalized for suicidal thoughts. Because of the presence of intensive and all-encompassing interests, which she pursued with energy, a diagnosis of bipolar disorder was erroneously formulated, and she was treated with Lithium, Aripiprazole and Asenapine without substantial changes. She was then referred to an outpatient clinic and therapy was switched to Vortioxetine and subsequently Lurasidone, Valproic acid and Quetiapine, with no substantial benefit. Only in 2020 she was diagnosed with ASD at the “Autism Lab” at the University of Pavia, Italy, which is an outpatient service dedicated to the diagnosis of ASD in adulthood. Diagnosis of ASD was made according to ADOS-2 (Communication + social interaction 7; cut off score for ASD ≥ 7) and ADI-R scores (Qualitative Abnormalities in Reciprocal Social Interaction 14 - cut-off > 10; Qualitative Abnormalities in Communication 5 - cut-off > 8; Restricted, Repetitive, and Stereotyped Patterns of Behavior 7 - cut-off > 3) as well as to clinical judgment of a senior psychiatrist. Severity of autistic symptoms was moderate (Social Responsiveness Scale total score=93). A comorbid diagnosis of depression was made using the MINI interview and she was treated with Fluvoxamine up to 200 mg daily with no change in symptoms. She was then switched to Sertraline up to 200 mg daily with only a slight reduction in depressive symptoms.

In 2021, as she did not achieve remission from depressive symptoms, she was started intranasal esketamine at the Community Mental Health Service of Pavia. From week 1 to 4 she was administered esketamine 84 mg twice weekly; from week 5 to 8 she received esketamine 84 mg once a week. To date, she still receives 84 mg esketamine twice monthly. The following evaluation scales were administered before the first drug administration (T0) and repeated after one week (T1), four weeks (T2) and eight weeks (T3) of treatment: Montgomery Asberg Depression Rating Scale (MADRS), Columbia-Suicide Severity Rating Scale (C-SSRS), Dissociative Experiences Scale (DES-II), Psychache Scale (PSA), Reading the Mind in the Eyes Test (RMET). 

**Figure 1:**
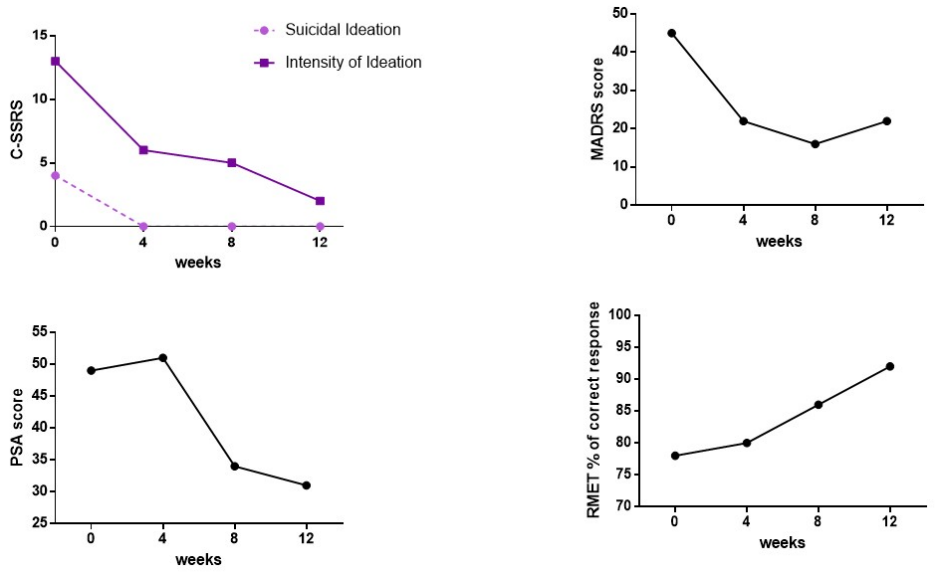
C-SSRS, MADRS, PSA total scores and RMET percentage of correct responses at baseline and at the different follow-ups

No side effects were reported and vital parameters during administration were always stable. MADRS total scores decreased progressively, as well as PSA scores (Figure 1(Fig. 1)). Suicidal ideation measured with the C-SSRS immediately disappeared without any relapse (Figure 1(Fig. 1)). Social cognition, assessed by means of the RMET, showed a slight increased, probably due to a learning effect (Figure 1(Fig. 1)). Of note, accuracy in emotion recognition was in normal range even at the beginning of the administration. G's subjective experience of depressive symptoms seems also improved. 

## Conclusion

This case report suggests that intranasal esketamine may be efficacious and safe in the treatment of resistant depression comorbid to ASD. This is particularly relevant as therapeutic options in treating depression in ASD are limited and often with moderate efficacy. Additionally, as ASD subjects usually experience more side effects than typically developing individuals, our results are promising.

Our experience could pave the way for further studies in the form of double blind, randomized controlled trials, aimed at investigating the role of intranasal esketamine not only in resistant depression alone but also in comorbid psychiatric conditions.
